# The cGAS-STING pathway: a therapeutic target in chromosomally unstable cancers

**DOI:** 10.1038/s41392-023-01328-4

**Published:** 2023-01-30

**Authors:** Haiyan Yan, Weiguo Lu, Fangwei Wang

**Affiliations:** 1 Key Laboratory of Novel Targets and Drug Study for Neural Repair of Zhejiang Province, School of Medicine, Hangzhou City University, 310015 Hangzhou, China; 2 Women’s Hospital, Zhejiang University School of Medicine, 310006 Hangzhou, China; 3 Cancer Center, Zhejiang University, 310058 Hangzhou, China; 4 The MOE Key Laboratory of Biosystems Homeostasis & Protection and Zhejiang Provincial Key Laboratory of Cancer Molecular Cell Biology, Life Sciences Institute, Zhejiang University, 310058 Hangzhou, China

**Keywords:** Cancer therapy, Cell biology

In a recent study published in *Nature*, Hong et al. revealed that the cyclic GMP-AMP synthase (cGAS)-stimulator of interferon genes (STING) innate immune pathway is critically required for the IL-6-dependent survival of chromosomally unstable cancer cells,^1^ implying that chronic inflammation can be therapeutically targeted to kill cancers displaying chromosomal instability (CIN).

CIN, usually defined as a persistently elevated rate of gain or loss of whole chromosomes during mitotic cell division, can also cause structural alterations of chromosome fragments. Representing a hallmark of cancer, CIN contributes to tumorigenesis through generating intratumoral genomic heterogeneity that drives cancer evolution and causes poor patient prognosis.^2^ It is therefore of pivotal importance to identify novel strategies to treat cancers with CIN.

CIN often results in the formation of micronuclei containing whole chromosomes or fragments of chromosomes which are not incorporated into the daughter nuclei. Micronuclei are susceptible to nuclear envelope collapse, which exposes chromosomal double-stranded DNA (dsDNA) to the cytoplasmic environment, initiating a cGAS-STING-dependent inflammatory immune response.

cGAS-STING was originally discovered as a sensor of viral DNA,^3–5^ whose activation normally facilitates proinflammatory signaling controlled by type I interferon (IFN), which is expected to promote apoptosis and immunosurveillance. Strikingly, Hong et al. found that IL-6-STAT3 signaling axis downstream of cGAS-STING instead enables the survival of cancer cells with CIN.^1^

The authors employed two well-established strategies to induce acute and chronical CIN phenotypes. Acute CIN was induced by treating cells with small-molecule inhibitors of the cell cycle kinases Mps1 and Wee1, which cause a strong increase in chromosome missegregation and the formation of micronuclei. Chronic CIN was induced in cells and mice by overexpressing a dominant-negative mutant of the microtubule depolymerase MCAK that causes chromosome missegregation.

It was first showed in human BT549 triple-negative breast cancer (TNBC) cells and 4T1 mouse TNBC cells that acute CIN induction effectively activated the cGAS-STING signaling, resulting in the increased phosphorylation of STAT1, STAT3, and IRF3. Interestingly, CRISPR-Cas9-mediated knockout of cGAS or STING, or treatment with a small-molecule inhibitor of cGAS, strongly reduced the viability of cells with acute CIN, indicating that cGAS-STING signaling enables the tolerance of TNBC cells to acutely induced CIN. Moreover, knockout of cGAS or STING reduced cell proliferation following chronic induction of CIN, and decreased tumor growth in allografted mice with chronic CIN. Thus, functional cGAS-STING signaling pathway promotes survival of cancer cells with CIN in vitro and in vivo.

Then Hong et al. investigated the key signaling pathways downstream of CIN-activated cGAS. Transcriptomic analysis in TNBC cells showed that cGAS knockout reduced the CIN-triggered increase of IFN-STAT1-STAT3 signaling and TNF-NF-кB signaling. Functional genetic analysis showed that knockout of STAT3, but not STAT1, sensitized cells to acute or chronic CIN induction, indicating the importance of STAT3 activation, but not STAT1 activation, for the survival of chromosomally unstable cells downstream of cGAS.

The authors further found that knockout of STAT1, but not STAT3, largely rescued the sensitivity of cGAS knockout cells to acute and chronic CIN induction. Moreover, while overexpressing a constitutively active mutant of STAT1 increased the sensitivity to acute CIN induction, STAT1 knockout eliminated CIN-mediated death of STAT3 knockout cells. Thus, STAT1 activation/phosphorylation promotes the death of cells with CIN.

Next, the authors examined the contribution of RELA-mediated canonical and RELB-mediated non-canonical NF-кB signaling to CIN sensitivity. The results showed that knockout of RELB or its activator NIK, but not RELA, sensitized cells to acute CIN induction. Thus, the inflammatory signaling induced by CIN has opposing effect on cell survival, i.e., the viability of cells with CIN requires cGAS-STING-STAT3 and non-canonical NF-кB signaling that antagonizes cell death caused by STAT1 activation.

Further transcriptomic analysis showed that IL-6, an inflammatory cytokine required for the activation of STAT3 signaling, was upregulated upon acute CIN induction in a cGAS, STING and RELB-dependent manner. Interestingly, following CIN induction, supplementation with recombinant IL-6, or lentiviral overexpression of IL-6, restored the viability of cGAS knockout and STING knockout cells. The observation that IL-6 supplementation did not restore the viability of STAT3 knockout cells experiencing CIN indicated that IL-6 acts upstream of STAT3. Thus, the survival of TNBC cells with acute CIN relies on a cGAS-STING-mediated IL-6-STAT3 pathway.

Strikingly, treatment with tocilizumab, a humanized monoclonal antibody that inhibits IL-6-STAT3 pathway through binding the IL-6 receptor (IL-6R), significantly reduced the viability and proliferation of TNBC cell lines with acute or chronic CIN, but had no effect on cells without CIN induction, or on untransformed breast epithelia cells. Then the authors xenografted TNBC cells with chronic CIN into immunocompromised mice, and found that tocilizumab treatment strongly reduced the growth of tumors with high rate of CIN. Similarly, tocilizumab treatment in syngeneic allografts largely delayed the progression of tumors with chronic CIN. Thus, pharmacological inhibition of IL-6R by tocilizumab shows selective toxicity to TNBC cells undergoing acute or chronic CIN, and selectively blocks the in vivo outgrowth of human and mouse TNBC cells with CIN.

Hong et al. further performed correlation analysis of datasets from human primary breast cancers in The Cancer Genome Atlas, and found that tumors with high cGAS expression had high CIN, as well as elevated expression of IL-6/IL-6R. Further analysis showed that, compared to tumors with high overall inflammation, tumors with high CIN and activated IL-6 signaling had reduced patient survival. Thus, cGAS-IL-6-IL-6R signaling is frequently activated in human breast cancers, resulting in poor patient prognosis.

Moreover, gene essentiality analysis using the DepMap database showed that high IL-6R level is associated with less dependency on genes involved in the regulation of DNA repair and chromosome segregation. Next, the authors induced chronic or acute CIN in a panel of non-TNBC cell lines of different types with different expression levels of IL-6 and IL-6R. They found that, following treatment with increasing doses of tocilizumab, only cancer cell lines expressing high levels of IL-6 and/or IL-6R were susceptible to tocilizumab-induced cell death. Moreover, in a lung cancer xenograft mouse model, treatment with tocilizumab significantly delayed the growth of tumor with high but not low rates of CIN. In addition, tocilizumab treatment largely reduced the viability of primary cell cultures with high IL-6/IL-6R expression, which were derived from genetically engineered mouse model of CIN-induced acute T-cell lymphoma.

**Fig. 1:**
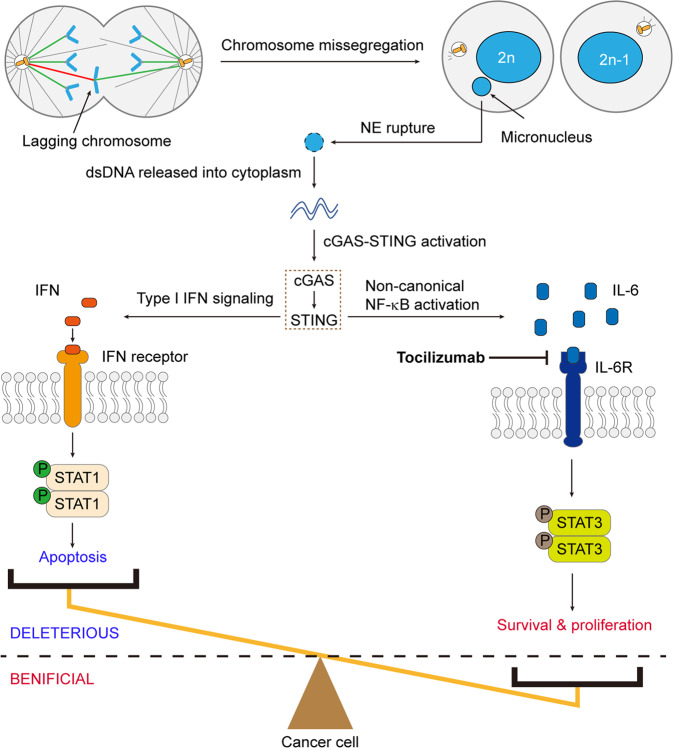
The cGAS-STING pathway drives IL-6-STAT3 signaling to enable survival of chromosomally unstable cancers. The viability of cancer cells with CIN relies on the balance between STAT1 and STAT3 signaling downstream of cGAS-STING. Inhibition of IL-6 signaling by the clinically used blocking antibody tocilizumab selectively reduces the survival of cancer cells experiencing CIN

Taken together, Hong et al. revealed that the cGAS-STING axis drives IL-6 signaling to enable the survival of cancers with CIN (Fig. 1). They showed that CIN induces cGAS-STING which subsequently increases IL-6 expression and activates STAT3 signaling, thereby promoting cancer cell survival and growth. Their study not only explains why loss-of-function mutations in genes encoding cGAS and STING are rarely found in primary tumors, but also uncovers a highly selective and targetable vulnerability of chromosomally unstable cancers.

Future studies are required to investigate, at the mechanistic level, how cancers with frequent chromosome missegregation cope with cGAS-STING signaling pathway. Clinical trials are also anticipated to repurpose tocilizumab, a clinically approved drug used for the treatment of autoimmune diseases such as arthritis, to improve the prognosis of patients with cancers displaying CIN and activated IL-6-STAT3 signaling. Moreover, since CIN-inducing spindle poisons such as paclitaxel and vincristine are widely used for chemotherapy, it will be of great interest to test whether they can synergize for cancer therapy with tocilizumab.
